# Routine on-table cholangiography during cholecystectomy: a systematic review

**DOI:** 10.1308/003588412X13373405385331

**Published:** 2012-09

**Authors:** MS Sajid, C Leaver, Z Haider, T Worthington, N Karanjia, KK Singh

**Affiliations:** ^1^Western Sussex Hospitals NHS Trust,UK; ^2^Royal Surrey County Hospital NHS Foundation Trust,UK

**Keywords:** Gallstones, Common bile duct stones, Cholecystectomy, On-table cholangiography

## Abstract

**INTRODUCTION:**

The aim of this review was to systemically analyse trials evaluating the efficacy of routine on-table cholangiography (R-OTC) versus no on-table cholangiography (N-OTC) in patients undergoing cholecystectomy.

**METHODS:**

Randomised trials evaluating R-OTC versus N-OTC in patients undergoing cholecystectomy were selected and analysed.

**RESULTS:**

Four trials (1 randomised controlled trial on open cholecystectomy and 3 on laparoscopic cholecystectomy) encompassing 860 patients undergoing cholecystectomy with and without R-OTC were retrieved. There were 427 patients in the R-OTC group and 433 patients in the N-OTC group. There was no significant heterogeneity among trials. Therefore, in the fixed effects model, N-OTC did not increase the risk (*p*=0.53) of common bile duct (CBD) injury, and it was associated with shorter operative time (*p*<0.00001) and fewer peri-operative complications (*p*<0.04). R-OTC was superior in terms of peri-operative CBD stone detection (*p*<0.006) and it reduced readmission (*p*<0.03) for retained CBD stones.

**CONCLUSIONS:**

N-OTC is associated with shorter operative time and fewer peri-operative complications, and it is comparable to R-OTC in terms of CBD injury risk during cholecystectomy. R-OTC is helpful for peri-operative CBD stone detection and there is therefore reduced readmission for retained CBD stones. The N-OTC approach may be adopted routinely for patients undergoing laparoscopic cholecystectomy providing there are no clinical, biochemical or radiological features suggestive of CBD stones. However, a major multicentre randomised controlled trial is required to validate this conclusion.

Common bile duct (CBD) injury is a known complication following open as well as laparoscopic cholecystectomy. The incidence of CBD injury after laparoscopic cholecystectomy has been reported as 0.2–0.4%, which is still somewhat higher than for long-time traditional open cholecystectomy (0.16–0.2%).^1–5^ Several risk factors for CBD injury during cholecystectomy have been reported. Prominent risk factors include acute cholecystitis, acute biliary pancreatitis, bleeding in Calot’s triangle, ‘shrunken’ gallbladder, impacted stones in the Hartmann’s pouch, aberrant extrahepatic biliary channels anatomy and aberrant biliary vasculature.^6–9^ In addition, iatrogenic CBD injury in experienced hands has also been reported to contribute in more than 50% of cases in the presence of one or more risk factors.^6,10–12^

Routine on-table cholangiography (R-OTC) during cholecystectomy has been advocated to reduce CBD injury by better delineation of biliary channels anatomy and helps to devise strategies for the management of co-existent CBD stones. Controversies still exist about the exact indications for OTC as an adjunct to laparoscopic cholecystectomy or laparoscopic converted to open cholecystectomy. Advocates of R-OTC propose the theory of reduced CBD injury due to fine anatomical delineation of the cystic duct, common hepatic duct and CBD.^13–15^ However, opponents of R-OTC contend that the routine use of this procedure during cholecystectomy does not offer enhanced clinical utility and it is responsible for prolonged operative time. R-OTC as an adjunctive procedure at the time of cholecystectomy is also responsible for the increased overall cost.^16–19^

The objective of this review was to systemically analyse the published randomised controlled trials evaluating the efficacy of R-OTC versus no on-table cholangiography (N-OTC) in patients undergoing laparoscopic, laparoscopic converted to open and open cholecystectomy using the principles of meta-analysis.

## Methods

Relevant prospective randomised controlled trials on R-OTC during cholecystectomy until April 2011 were included in this review. The Cochrane Hepato-Biliary Group Controlled Trial Register, the Cochrane Central Register of Controlled Trials, MEDLINE® and Embase™ were searched until April 2011 using the MeSH (Medical Subject Headings) keywords ‘cholecystectomy’ and ‘on-table cholangiography’. These headings were searched independently and also used in combination with ‘laparoscopic surgery’ and ‘cholangiography’. A filter recommended by the Cochrane Collaboration^20^ was used to filter out irrelevant studies in MEDLINE® and Embase™. The references of the studies found were searched to identify further trials. Studies analysing the role of OTC in open cholecystectomy, laparoscopic cholecystectomy and laparoscopic converted to open cholecystectomy were included in this review. In addition, studies publishing data on readmission as a consequence of retained CBD stones were also included.

Two authors (MSS and CL) independently identified the relevant studies, extracted data related to the outcomes and secured the data on an Excel® spreadsheet (Microsoft, Redmond, WA, US). These were further confirmed by the third author (ZH). Any conflict about data was resolved by mutual agreement among the authors. The software package RevMan 5.0.1 (Nordic Cochrane Centre, Copenhagen, Denmark) was used for analysis. The odds ratio (OR) with 95% confidence interval (CI) was calculated for binary data variables and the mean difference (MD) with a 95% CI was calculated for continuous data variables. If the mean values were not available for continuous outcomes, median values were used for the purpose of meta-analysis. If the standard deviation was not available, it was calculated according to the guidelines of the Cochrane Collaboration.^20^ This involved assumptions that both groups have the same variance, which may not be true.

The random effects model^21^ and the fixed effects model^22^ were used to calculate the combined outcome for both binary and continuous variables. In case of heterogeneity, only the results of the random effects model were reported. Heterogeneity was explored using the chi-squared test, with significance set at *p*<0.05, and quantified^23^ using I^2^, with a maximum value of 30% identifying low heterogeneity.^20^

The Mantel–Haenszel method was used for the calculation of the OR under the fixed effects as well as the random effects model.^24^ In a sensitivity analysis, 0.5 was added to each cell frequency for trials in which no event occurred in either the treatment or control group, according to the method recommended by Deeks *et al*.^25^ The estimate of the difference between both techniques was pooled depending on the effect weights in results determined by each trial estimate variance. The forest plot was used for the graphical display of results from the meta-analysis. The square around the estimate stands for the accuracy of the estimation (sample size) and the horizontal line represents the 95% CI.

## Results

**Figure 1:**
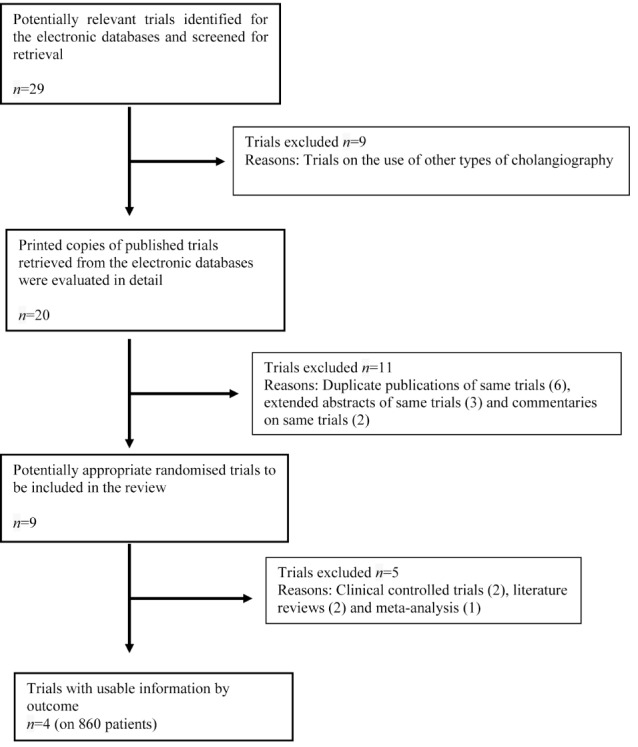
Trial selection methodology

Figure 1 explains the study methodology and literature search. Four studies encompassing 860 patients undergoing a cholecystectomy with either R-OTC or N-OTC were retrieved from the electronic databases.^26–29^ There were 427 Figure 1 Trial selection methodology patients in the R-OTC group and 433 patients in the N-OTC group. One included trial involved patients undergoing an open cholecystectomy^26^ and the remaining three trials were conducted on patients undergoing a laparoscopic and/or a laparoscopic converted to open cholecystectomy.^27–29^ The recruited patients in the included randomised trials did not have clinical, biochemical or radiological evidence of CBD stones pre-operatively. However, a criterion to rule out pre-operative CBD stones among included studies was not homogenous. The characteristics of these trials are given in Table 1. The variables used to achieve a combined outcome are given in Table 2.

**Table 1:** Characteristics of included trials

Trial	Type of trial	Country	Surgical procedure	Comparison groups	Follow-up duration
Hauer-Jensen* et al*, 1986^26^	RCT	Norway	Open cholecystectomy	R-OTC vs N-OTC	1 year
Soper and Dunnegan, 1992^27^	RCT	US	Laparoscopic cholecystectomy	R-OTC vs N-OTC	1 year
Nies* et al*, 1997^28^	RCT	Germany	Laparoscopic cholecystectomy	R- OTC vs N-OTC	1 year
Khan* et al*, 2011^29^	RCT	UK	Laparoscopic cholecystectomy	R-OTC vs N-OTC	1 year

RCT: randomised controlled trial; R-OTC = routine on-table cholangiography; N-OTC = no on table-cholangiography

**Table 2:** Outcome variables

Trial	Number of patients	Operative time	CBD stones detection	CBD injury	Complications	Readmission
Hauer-Jensen* et al*, 1986^26^						
R-OTC	142	81.4 (75.9–86.9)	4	0	21	0
N-OTC	138	58.1 (53.7–62.5)	0	0	8	0
Soper and Dunnegan, 1992^27^						
R-OTC	56	94 ±3	3	0	1	0
N-OTC	59	78 ±3	0	0	0	0
Nies* et al*, 1997^28^						
R-OTC	138	92 ±31	3	0	10	0
N-OTC	137	77 ±28	0	0	8	5
Khan* et al*, 2011^29^						
R-OTC	91	66 ±2	3	0	1	0
N-OTC	99	54 ±3	0	1	2	4

CBD = common bile duct; R-OTC = routine on-table cholangiography; N-OTC = no on table-cholangiography

### Methodological quality of included studies

The methodological quality of included trials was assessed by Jadad *et al* and Chalmers *et al*.^30,31^ All trials were of moderate to good quality. The Mantel–Haenszel fixed effects model was used to compute robustness and susceptibility to any outlier among these trials. The allocation concealment and blinding of investigator/assessor was not reported clearly. There was no statistically significant heterogeneity (clinical and methodological diversity) among trials except in the case of operative time.

### Operative time

**Figure 2:**
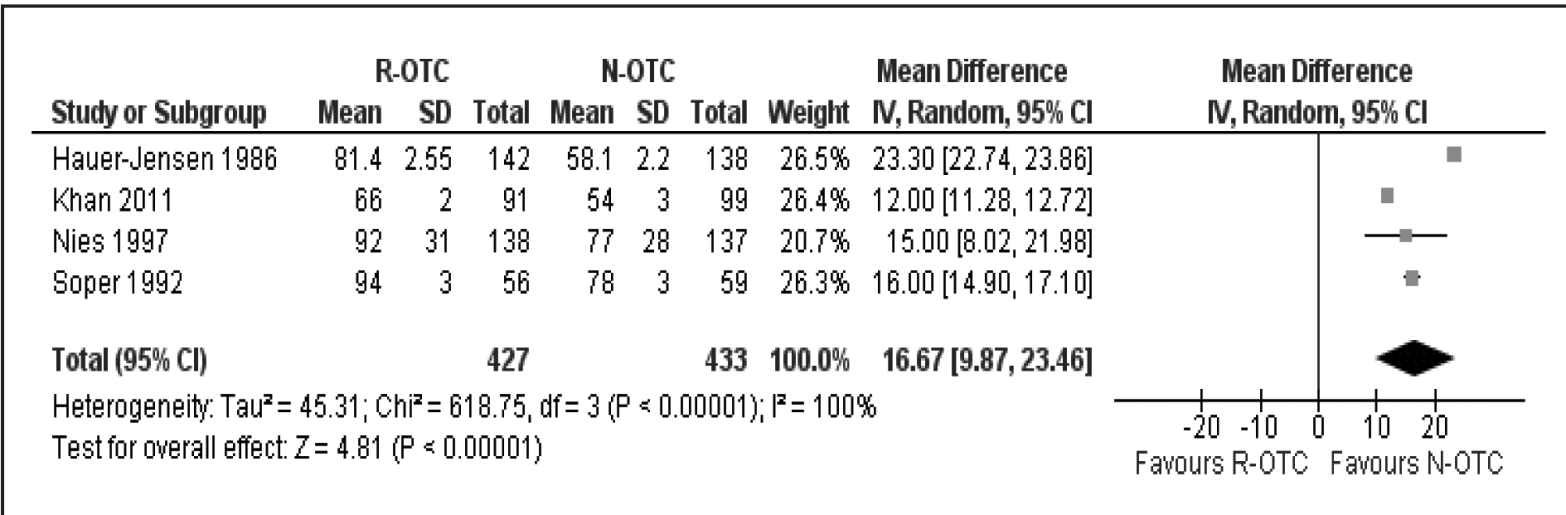
Operative time

There was a significant heterogeneity (τ^2^=45.31; X^2^=618.75; df=3; *p*<0.00001; I^2^=100%) among the four trials. Therefore, in the random effects model, the operative time for the N-OTC group was shorter (MD: 16.67 minutes; 95% CI: 9.87–23.46 minutes; z=4.81; *p*<0.00001; Fig 2).

### Peri-operative biliary channels stone detection

**Figure 3:**
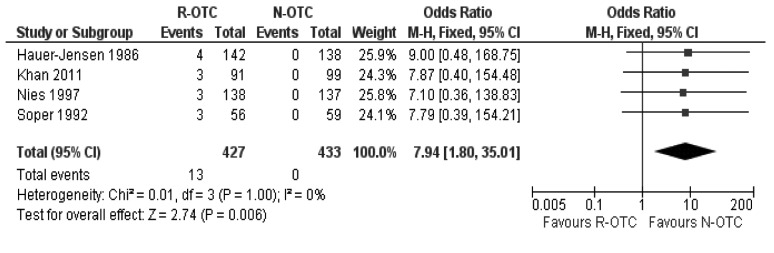
Common bile duct stone detection rate

There was no heterogeneity (X^2^=0.01; df=3; *p*=1.0; I^2^=0%) among the included trials. Therefore, in the fixed effects model, R-OTC was superior to N-OTC in terms of peri-operative CBD stone detection (OR: 7.94; 95% CI: 1.80–35.01; z=2.74; *p*=0.006; Fig 3) and thus guided the operating surgeon in devising a strategy for the management of CBD stones.

### Incidence of CBD injury

**Figure 4:**
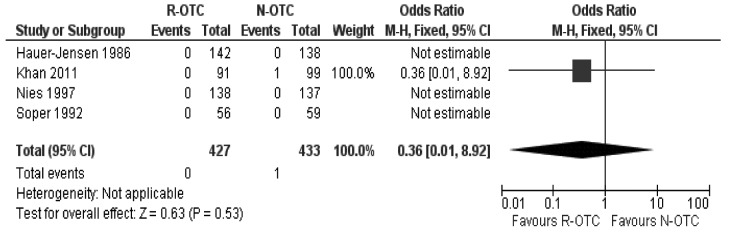
Incidence of common bile duct injury

In the fixed effects model, incidence of CBD injury following cholecystectomy was statistically equivalent between the two groups and N-OTC did not increase the risk of CBD injury (OR: 0.36; 95% CI: 0.01–8.92; z=0.63; *p*=0.53; Fig 4).

### Peri-operative complications

**Figure 5:**
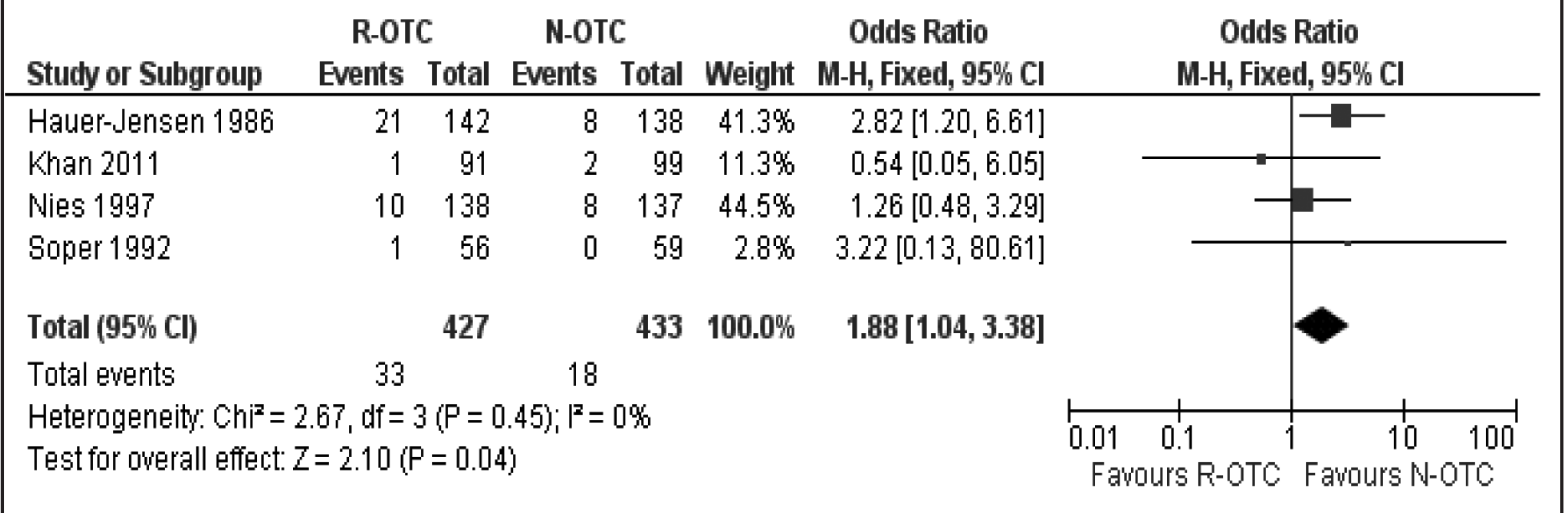
Peri-operative complications (all trials)

There was no heterogeneity (X^2^=2.67; df=3; *p*=0.45; I^2^=0%) among the trials. Therefore, in the fixed effects model, the risk of peri-operative complications was higher following R-OTC (OR: 1.88; 95% CI: 1.04–3.38; z=2.10; *p*=0.04; Fig 5).

### Readmission rate secondary to retained CBD stones

**Figure 6:**
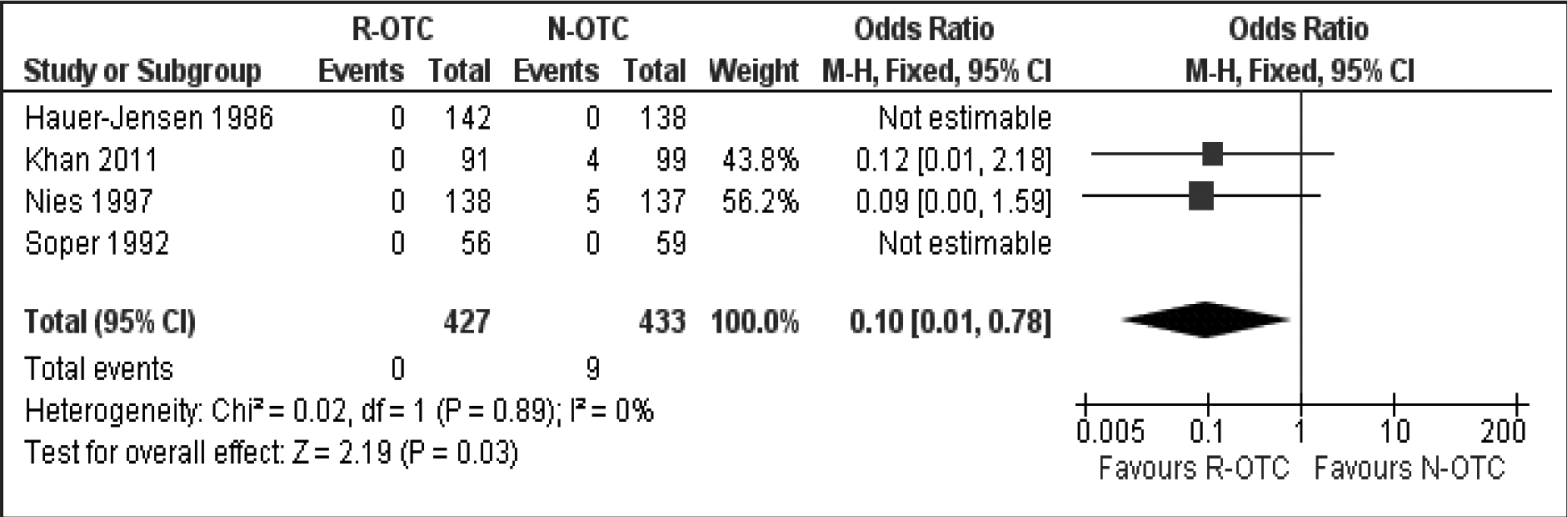
Readmission rate

There was no heterogeneity (X^2^=0.02; df=1; *p*=0.89; I^2^=0%) among the trials. Therefore, in the fixed effects model, the risk of readmission for retained CBD stones was lower for the R-OTC group compared with patients receiving N-OTC (OR: 10; 95% CI: 0.01–0.78; z=2.19; *p*=0.03; Fig 6).

### Subgroup analysis

**Figure 7:**
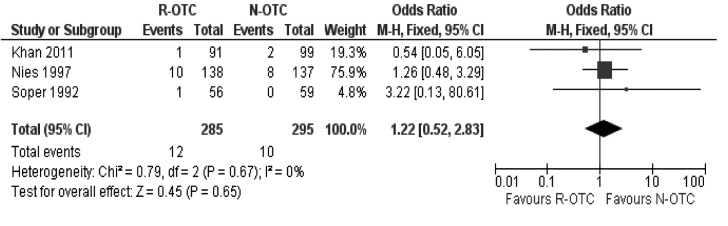
Peri-operative complications (trials on laparoscopic cholecystectomy only)

Subgroup analysis of the randomised trials on laparoscopic cholecystectomies only showed that R-OTC was associated with a longer operative time, and equivalent CBD injury incidence and peri-operative complications (Fig 7) compared with N-OTC. However, R-OTC was found to be slightly superior to N-OTC in terms of CBD stone detection rate during surgery and readmission rate secondary to retained CBD stones.

## Discussion

In order to avoid CBD injury and other operative complications, visualisation of biliary anatomy during cholecystectomy has long been an attractive as well as challenging task for surgeons. Since the introduction of OTC in 1932 by Mirizzi,^32,33^ surgeons all over the world have been divisive about its routine use, selective use or no use at all. While this debate is still going on fervently, other techniques to delineate biliary tree anatomy are also being investigated and reported in the medical literature. These relatively innovative techniques include fluorescent cholangiography,^34^ intra-operative digital cholangiography,^35^ intravenous cholangiography,^36^ laparoscopic ultrasonography^37^ and pre-operative three-dimensional computed tomography cholangiography.^38^ These approaches have shown some promising results but, being simple, technically less challenging and economically cost effective, OTC is still probably the most commonly used and investigated adjunctive procedure for cholecystectomy.

Use of R-OTC during cholecystectomy has been reported to reduce CBD injury^39^ but does not abolish this risk completely.^14^ There has been significant contradiction in the reported results on the effect of R-OTC in terms of CBD injury during laparoscopic cholecystectomy.^11,12,40,41^ Archer *et al* claimed 81% of CBD injuries were detected when R-OTC was performed with laparoscopic cholecystectomy while only 45% of CBD injuries were diagnosed clinically without R-OTC.^11^ In contrast, other authors reported that R-OTC does not influence the CBD injury detection rate^2^ and the misinterpretation rate of R-OTC has also been quoted as being significantly higher.^8,40^ A study published in 2011 on 31,838 patients undergoing a laparoscopic cholecystectomy reported equivalent chances of missing CBD injury with and without R-OTC.^12^

The incidence of CBD stones in low risk patients is around 1.7%, a risk that does not warrant R-OTC.^19^ Therefore, the group of patients ranging from low risk to high risk for choledocholithiasis may be stratified^19^ pre-operatively for endoscopic retrograde cholangiopancreatography instead of performing adjunctive R-OTC at the time of cholecystectomy.

R-OTC may result in false positive rates of 2.1% to as high as 67%.^42,43^ The positive predictive value ranges from 63% to 92%, causing a significant number of patients to have an unnecessary additional procedure,^44^ which leads to increased operative morbidity and mortality.^45^ In addition, R-OTC may itself cause CBD injury, possibly due to manipulation of the cystic duct and sometimes leading to its complete transaction of the CBD.^46^

The financial implications of R-OTC should be considered in this era of economic downturn. The estimated cost of diagnosing one CBD stone in the presence of mild risk of choledocholithiasis has been reported as around $80,000^47^ and the cost of detection of one unsuspected but clinically significant CBD stone was $166,500.^48^ In a series of 500 patients undergoing laparoscopic cholecystectomy, only 1 patient was readmitted with a retained CBD stone over 2–16 years of follow-up.^42^ Sixty per cent of CBD stones that become symptomatic do so within eighteen months of cholecystectomy and therefore only a small minority of unsuspected CBD stones are clinically relevant.^49^

## Conclusions

Based on this review, N-OTC is associated with shorter operative time and fewer peri-operative complications. It is comparable with R-OTC in terms of CBD injury risk during cholecystectomy. R-OTC is helpful for peri-operative CBD stone detection and consequently reduces the readmission rate for retained CBD stones. For patients undergoing a cholecystectomy, the N-OTC approach may therefore be adopted routinely, provided there is no clinical, biochemical or radiological evidence of CBD stones.

To our knowledge, this is the first systematic review on the effectiveness of R-OTC during cholecystectomy. We are aware that the included trials are statistically of moderate quality due to the lack of masking/blinding, the absence of intention-to-treat analysis and weak power calculations. This review contains the analysis of 860 patients only, which reflects merely a very small percentage of cholecystectomies and R-OTCs performed worldwide. This conclusion may therefore be considered weak and biased. Hence, a major multicentre randomised controlled trial is required to validate this conclusion.
